# The Postoperative Analgesic Effect of Morphine and Paracetamol in the Patients Undergoing Laparotomy, Using PCA Method

**DOI:** 10.5539/gjhs.v6n1p207

**Published:** 2013-12-20

**Authors:** Siamak Yaghoubi, Reza Pourfallah, Ameneh Barikani, Hamid Kayalha

**Affiliations:** 1 Anesthesiology Department, Velayate Hospital, Qazvin University of Medical Sciences, Qazvin, Iran; 2 Community Medicine Department, Faculty of Medicine, Qazvin University of Medical Sciences, Qazvin, Iran

**Keywords:** paracetamol, morphine, pain, surgery, laparotomy, PCA, analgesic

## Abstract

**Objective:**

postoperative pain increases the activity of the sympathetic system, causes hypermetabolic conditions, retains salt and water, increases glucose, fatty acid lactate and oxygen consumption, weakens the immunity system which delays wound healing.

Our object was comparison of the analgesic effect of morphine and paracetamol in the patients undergoing laparotomy, using PCA method.

**Method:**

Seventy patients who had undergone laparotomy were studied using double blind randomized clinical trial (35 patients received morphine and 35 paracetamol) in the Shahid Rajaee Center and Velayat Hospital (Qazvin, Iran). People using opioids, painkillers and sedatives regularly and in large doses and patients with a history of lung or liver problems did not participate in this project. The parameters of the severity of pain and nausea (VAS), hemodynamic changes (BP and HR), pruritus, arterial oxygen desaturation and patient satisfaction (VAS) of both groups were measured by a third party (trained colleague). The data was analyzed using SPSS 16 statistical software then descriptive results were extracted and ultimately the groups were compared using the following statistical tests: student’s T-test, chi 2 and Fisher’s exact test (P<0.05).

**Findings:**

The mean age of the participants was 45±12.5 years. Women constituted 24.3% of the patients and men 75.7%. The average pain severity for morphine and paracetamol groups (VAS) was 5.3±2.2and 6.37±1.7 after2 hours and reached 1.91±1.3 and 2.49±1.3 after 8 hours (after the operation) respectively. There was a significant difference between the groups after 2 and 4 hours in terms of pain severity (after 2 hours P=0.007 and after 4 hours P=0.047). However there was no significant difference between the average pain severity of the studied groups (after 6 hours P=0.4 and 8 hours P=0.08).

After 8 hours, the average nausea severity was the minimum in both groups being 1.71±1.6 and 1.43±1.1 in morphine and paracetamol groups respectively. Nausea severity was higher after 2 hours in paracetamol group. In morphine group, it was higher after 4, 6 and 8 hours. Difference between the groups was not significant. The average satisfaction level (VAS) for morphine and paracetamol groups reached from 5.29±2.3 and 4.2±2.4 after 2 hours, to 7.94±1.8 and 7.69±2.1 after 8 hours (after the operation), respectively. The average satisfaction level of patients was higher in morphine group in 2,4,6 and 8 hours and except for, after 4 hours (P=0.01), the satisfaction difference between both groups was not significant in other hours (P=0.06 after 2 hours, P=0.6 after 6 hours and P=0.5 after 8 hours)

**Conclusion:**

Morphine seems to be more effective at 2 and 4 hours, but after 4 hours they have similar effects, the satisfaction difference between both groups was not significant in the patients.

## 1. Introduction

Reducing postoperative pain, especially with some specific analgesic regimens, reduces perioperative mortality (Miller, Erikson, Fleisher, Wiener-Kronish, & Young, 2010).

Transmission of painful stimuli to the CNS (Central Nervous System) creates neuro-endocrine stress responses and increases local and systemic inflammatory substances. Neuroendocrine responses to pain include increase in the secretion of catecholamines and catabolic hormones such as cortisol, glucagon, aldosterone, morphine, angiotensin, ACTH (Adreno-corticotropic hormone) and ADH (antidiuretic hormone), and decrease in anabolic hormones which results in salt and water retention, blood glucose, free fatty acids, ketone bodies and lactate increase. The responses ultimately create a hyper-metabolic and catabolic state. Stress response increases postoperative coagulability caused by the decrease in anticoagulant levels, and increases pro-coagulants and ultimately increases DVT (Deep Vein Thrombosis) incidence and vascular and myocardial ischemia graft rejection (Miller et al., 2010).

On the other hand, stress response causes immune-suppression relative to the severity of surgical trauma which leads to hyperglycemia and reduced wound healing. Increase in catecholamines, due to uncontrolled pain, results in increased myocardial oxygen consumption, coronary artery disease, ischemia and myocardial infarction. Also stimulation of the sympathetic system delays the return of gastrointestinal motility and causes ileuses (Miller et al., 2010).

Pain reduces deep breath and cough which causes respiratory complications. Depending on the type of surgery, postoperative chronic pain can be seen in 10% to 65% of the patients. Uncontrolled acute postoperative pain is an important prognostic factor for the development of respiratory complications. Various drugs are used to control postoperative pain, among which narcotics, NSAID (Nonsteroidal_Anti-inflammatory Drugs), ketamine, topical anesthetics and paracetamol can be named. Side effects of narcotics include respiratory depression, nausea, drowsiness and drug tolerance and dependence (Miller et al., 2010; Knollman, Chabner, & Brunton, 2005; Waldman, 2010).

NSAID drugs, due to platelet dysfunction and the inhibition of thromboxane A2, reduce homeostasis. Additionally, they result in impaired renal function and side effects on bone repair and spinal fusion. Ketamines reduces central sensitization and prevent chronic pain. It also reduces 24-hour morphine usage with PCA (Patient controlled analgesia) method and has side effects such as nausea (Miller et al., 2010).

Topical anesthesics are used for nerve blocks (Miller et al., 2010). Paracetamol or acetaminophen or N-acetyl-p-aminophenol is an active metabolite of Phenacetin. It should be noted that Paracetamol could be a hepatotoxic but its chronic usage with a normal dose doesn’t result in liver dysfunction. It’s a good alternative particularly for the patients for whom aspirin is contraindicated such as patients suffering from peptic ulcer, aspirin allergy and children with febrile illnesses (Knollman et al., 2005). Nowadays, PCA method with morphine is recognized as a key element of postoperative analgesia. In this method analgesic drug can be injected by the patient himself. Drug prescription will be optimized and the pharmacodynamics and pharmacokinetics of the drugs will be reduced. The PCA device can be programmed in terms of bolus doses, the interval between doses and continuous infusion. Usually an optimal dose is determined which is neither too low nor too high to decrease side effects. Common intervals between the injections are 5 to 10 minutes.

PCA does not reduce the duration of hospitalization but provides shorter nursing time, higher satisfaction in the patients and improved pain and reduces lower respiratory depression. It is both as IV (intra venous) and epidural (Miller et al., 2010; Waldman, 2010; Rodgers, Webb, Stergios, & Newman, 1998).

With this technique receive of drug delay is prevented. It provides the highest flexibility for drug regulation and the best compatibility with the patients’ physiological differences. It is suitable for adults and children and is preferred to IM injection for postoperative pain control (Rodgers et al., 1998). It can be used for children older than 4 years and is preferred to IV, IM (intra muscular) and oral administration for all patients.

Morphine is the drug chosen for this method. Although morphine is standardized, it doesn’t seem preferable to other narcotics such as hydromorphone. It is also shown in some studies that infusion dose of narcotics causes nausea, drowsiness and hypoxemia and due to respiratory events it is necessary to monitor the patients (Miller et al., 2010). Due to the importance of postoperative pain control and because of the side effects of morphine, we decided to compare paracetamol with morphine in terms of their analgesic effectiveness and other side effects in this research.

## 2. Materials and Method

This research was done using double blind randomized clinical trial. This research was registered in IRCT (Iranian Registry of Clinical Trials) with number 2013012712298N1. The studied community includes 15 to 65 year-old patients with the ASA (American Society of Anesthesiologists) of 1 or 2, who had undergone laparotomy surgery. It was conducted in Shahid Rajaee Center and Velayat Hospital, on patients who underwent laparotomy surgery from February 2012 to October 2012. Seventy patients aging from 15 to 65 with the ASA of 1 and 2 underwent laparotomy surgery. The length of the operation was less than 2.5 hours, surgery time more than 2.5 hours was our exclusion criteria in our study. There was not significant difference between two groups (P>0.05). After being admitted, the patients were randomly divided into two groups with white and black cards. The morphine group received morphine with the dose of 0.02 mg/kg of their body weight. The maximum dose of paracetamol in a day was 3 grams. In our study we gave paracetamol 1.4mg/kg/hr. Two groups equal with regard to anesthesia methods. Men constituted 75.7% of the patients and women 24.3%. No significant gender difference was observed between the groups (P>0.05). Pain severity and side effects such as nausea, hemodynamic changes, pruritus, desaturation of arterial oxygen and patient satisfaction all using VAS(Visual Analog Scale), are measured by third party (trained colleague) for 8 hours, with 2 hour intervals. VAS is a scoring range system which is divided to 10 points and the patients rate their situation. One represents asymptomatic (Miller et al., 2010) and the 10 represents the most conceivable circumstances for the intended factor (Khan et al., 2007; Miller et al., 2010) which enables us to convert qualitative variables into quantitative ones. In this research, this scale has been used for pain, nausea and patient satisfaction. Respiratory complications are considered as decrease in arterial oxygen and its saturation below 90% in ambient air which was measured and monitored using pulse oximetry. By surgical laparotomy only cholecystectomy is meant. Hemodynamic changes are categorized as low (less than 20% change compared to the base blood pressure and heart rate), medium (change about 20% to 40% compared to the base blood pressure and heart rate) and high (more than 40% change compared to the base blood pressure and heart rate).

People using opioids, painkillers and sedatives regularly and in large doses and patients with a history of lung or liver problems did not participate in this project.

Ultimately the obtained data was statistically analyzed. The data obtained from both groups were inserted into the forms and the data such as pain (VAS), patient satisfaction (VAS), nausea (VAS), HR and BP changes, pruritus and respiratory complications of the patients using morphine and paracetamol were collected. Then the data was transferred to the computer using SPSS 16 software, descriptive findings were extracted and the groups were compared using chi-square, T-test and Fisher’s exact test with P<0.05.

## 3. Findings

Men constituted 75.7% of the patients and women 24.3%. Mean age of the patients of morphine and paracetamol groups were respectively 48±10.4 and 42±14.5 years old on average. No significant difference was observed in the age groups (P: 0.05).

**Figure 1:**
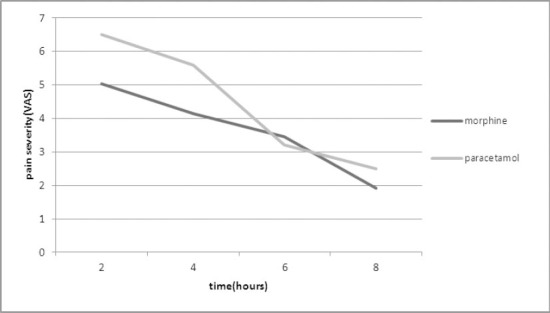
Comparative diagram of pain severity in both groups based on VAS scale

There was a significant difference between the groups after 2 and 4 hours after surgery in terms of pain severity (after 2 hour P=0.007 and 4 hours P=0.047) (Table 1) (Figure 1).

**Table 1:** Pain severity in morphine and paracetamol groups based on VAS scale with gender (N=70)

Mean pain severity	Morphine groups		P	Paracetamol groups		P	P(total)
	Male(n=35)	Female(n=35)		Male(n=35)	Female(n=35)		
**After 2 hours**	5.2±2.2	3.6±1.3	0.1	6.5±1.9	6±1.4	0.3	0.007
**After 4 hours**	4.3±2	3.2±1.6	0.2	5.3±1.9	4.4±1.4	0.1	0.047
**After 6 hours**	3.5±1.4	3±1.2	0.4	3.7±1.5	2.2±1.3	0.05	0.47
**After 8 hours**	2±1.4	1.4±0.5	0.3	2.8±1.3	1.7±0.9	0.05	0.08

Based on VAS scale, the highest and lowest pain severity in morphine and paracetamol groups was 9 and 1, respectively. The findings show that after the surgery the average pain severity in both groups decreases with the passage of time.

However there was no significant difference between the average pain severity of the studied groups (after 6 hours P=0.4 and 8 hours p=0.08).

**Figure 2:**
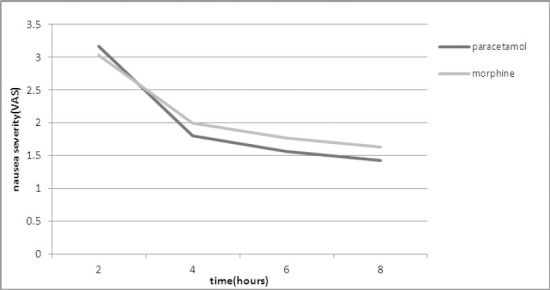
The comparative diagram of average nausea in both groups based on VAS scale

After 2 hours, the average nausea was 3.2±3.0 and 3.17±3 in morphine and paracetamol groups, respectively (Figure 2).

**Figure 3:**
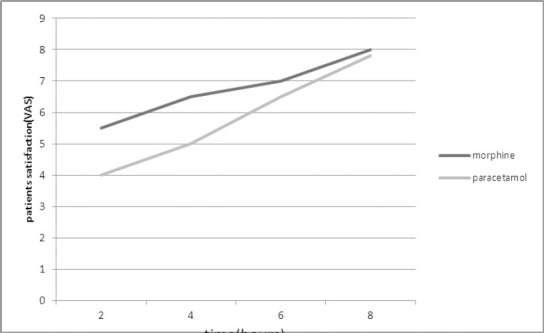
The comparative diagram of patients’ satisfaction level in both groups based on VAS scale

Nausea difference between the groups was not significant and the P value was higher than 0.05 in all cases. The average satisfaction level in both groups increased with the passage of time. The average satisfaction level of patients was higher in morphine group in all four data collection hours and except for four hours after (P=0.01) the satisfaction difference between both groups was not significant in other hours (P=0.06 after 2 hours, P=0.6 after 6 hours and P=0.5 after 8 hours) (Figure 3).

The highest and lowest satisfaction levels in morphine and paracetamol groups were 10 and 1, respectively. Throughout the study, HR change was not recorded higher than 40% in all patients (Table 2).

**Table 2:** The HR change of the patients in the studied groups (%) (Total number=70)

Drug	After 2 hours		After 4 hours		After 6 hours		After 8 hours	
	Low >20	Medium 20-40	Low>20	Medium 20-40	Low>20	Medium 20-40	Low>20	Medium 20-40
**Morphine (n=35)**	27 (77.1)	8 (22.9)	33 (94.3)	2 (5.7)	35 (100)	-	35 (100)	-
**Paracetamol (n=35)**	15 (42.9)	20 (57.1)	34 (97.1)	1 (2.9)	35 (100)	-	35 (100)	-
**P-value**	0.003		0.5		-		-	

After 2 hours, the HR change in the patients of paracetamol group was mostly between 20% to 40% (57.1%). This number for the most of morphine group was less than 20% (77.1%). The difference between morphine and paracetamol groups was significant (P=0.003).

After 4 hours, the HR change in more than 90% of the patients of both groups was less than 20% and the difference was not significant (P=0.5). In both groups the HR change was less than 20% at 6 and 8 hours. Throughout the study BP change was not more than 40% in all patients (Table 2).

**Table 3:** The BP change of the patients of the studied groups (%) (Total number=70)

Drug	After 2 hours		After 4 hours		After 6 hours		After 8 hours	
	Low>20	Medium 20-40	Low>20	Medium 20-40	Low>20	Medium 20-40	Low >20	Medium 20-40
Morphine (n=35)	29 (82.9)	6 (17.1)	34 (97.1)	1 (2.9)	35 (100)	-	35 (100)	-
Paracetamol (n=35)	16 (45.7)	19 (54.3)	35 (100)	-	35 (100)	-	35 (100)	-
P-value	0.001		0.5		-		-	

After 2 hours, the BP change in the patients of paracetamol group was mostly between 20% to 40% (54.3%). In morphine group, the BP change experienced by most patients was less than 20% (82.9%). The difference between the groups was significant (P<0.001 after 2 hours).

After 4 hours, the BP change in most patients in paracetamol group (97.1%) and all the patients in morphine group was less than 20%. The difference between the groups was not significant (P<0.5 after 4 hours). After 6 and 8 hours, the BP change was less than 20% for all the patients in both groups.

In this research the patients were studied after 2, 4, 6 and 8 hours in terms of pruritus and only one patient (2.9%) from the morphine group complained about pruritus after 2 hours, but it was not significant (P<0.5). There were no complaints about pruritus after 4, 6 and 8 hours.

The groups were also analyzed by pulse oximeter in terms of arterial oxygen desaturation and only 2 patients (5.7%) experienced arterial oxygen desaturation under 90% after 2 hours but the difference was not significant (P=0.2). After 4, 6 and 8 hours there was no report of arterial oxygen desaturation under 90%.

## 4. Discussion and conclusion

There are many studies conducted on postoperative analgesia using various drugs and methods in the past years. In the present study we compared morphine and paqracetamol analgesic effect.

Morphine had a stronger analgesic effect compared to paracetamol in the final results after 2 and 4 hours after surgery, but there was no significant different between them after 6 and 8 hours. There was no significant difference between the side effects of the drugs except for HR and BP after 2 hours and there was no significant difference in the satisfaction level of the patients after 2, 6 and 8 hours except for 4 hours which was higher in morphine group. In many studies researchers have found that 1g iv paracetamol used alone is just as effective as 30 mg ketorolac, 75 mg diclofenac or 10 mg morphine (Zhou, Tang, White, 2001; Van Aken, Thys, Veekman, & Buerkle, 2004). In this study, obviously paracetamol and morphine were more effective than placebo throughout the 10 hour study. No significant statistical and clinical difference was observed between morphine and paracetamol groups. The side effects in paracetamol groups were significantly lower than morphine group (P<0.027) (Van Aken et al., 2004). The amount of the drug used in our research and the aforementioned study was approximately equal and the only difference was in the method of morphine administration. In the aforementioned study, the administration method was IM but in our research PCA pomp was used. The results of both studies are generally similar and both find paracetamol comparable to morphine for postoperative pain control. With regard to the IV method used in our research to inject morphine compared to the IM method for the aforementioned research, morphine was more effective in the first hours compared to paracetamol. In the study of Arici, Gurbet, Türker, Yavaşcaoğlu and Sahin (2009), VAS scores were lower in the paracetamol group in the postoperative period and total morphine consumptions were lower in paracetamol group. In the research by Stoneham and Walters (1996) carried out to compare codeine and morphine using PCA method resulted in slight but not significant decrease in the pain severity of the patients. There was no clear difference in nausea severity and sedation level and RR between the groups. In the research by Remy, Marret and Bonnet (2005) adding acetaminophen to morphine only added 20% to the analgesic effect for 24 hours after the surgery. Many Studies have found that iv paracetamol has an opioid-sparing effect and by reducing the opioid requirement increases patient satisfaction (Hernández-Palazón, Tortosa, Martínez-Lage, & Pérez-Flores, 2001; Hynes, McCarroll, & Hiesse-Provost, 2006; Avellaneda et al., 2000).

In the research by Unlugenc, Vardar, and Tetiker (2008) conducted to compare postoperative analgesia and the side effects of morphine, pethidine and tramadol using IV-PCA method, all the three drugs were identical regarding pain severity and side effects; only in tramadol group higher amounts of fentanyl was needed to reduce the anesthetic function.

In the research by Stanley, Appadu, Mead and Rowbotham (1996) carried out to compare morphine and pethidine, there was no significant difference in sedation, nausea, pain relief, patient satisfaction and the need for anti-nausea drug between the groups.

In the research by Hyllested, Jones, Pedersen and Kehlet (2002) conducted to compare paracetamol and NSAID it was shown that NSAIDs are better than paracetamol for dental surgeries and they have identical effects for major and orthopedic surgeries. Although their combination is more useful than when they are used separately.

In another research conducted in 2007 by Inal, Celik and Tuncay to compare the effect of paracematol and meperidine on the patients needing elective caesarean section showed that the groups were very similar in terms of side effects. Ultimately in the above-mentioned study it was shown that IV paracetamol is clearly more effective than meperidine in pain control after cesarean section. In this research paracetamol was more effective than narcotics according to our investigations. The reason for the difference in the results could be the type of narcotics, method of administration, or surgery type but paracetamol was found useful in controlling pain in both studies.

Another research was conducted by Khan, Iqbal, Saleh, and Mohamed El Deek in 2007 on 84 patients who had undergone knee arthroscopy. The severity of pain in the patients was measured with VRS (*Verbal Rating Scale)* for 4 hours. It was similar in both groups but the severity of side effects such as nausea and dizziness were reported higher in morphine group.

Probably, the reason for the difference in side effects is the whole injection of narcotics compared to our research in which narcotics were administered to the patient gradually and within 24 hours. In the other hands many predictors of postoperative pain intensity or analgesic Consumption may be effect on pain after surgery such as gender, predictors of postoperative pain intensity and/or analgesic consumption.

Although In our study the distribution of gender was not equally but this different was not significant and therefore could not have a confounding effect. In systematic review that was conducted by Hui and et al was found that many studies could not show a significant correlation between gender and postoperative pain (Ip, Abrishami, Peng, Wong, & Chung, 2009; Kalkman et al, 2003; Mamie et al., 2004; Caumo et al., 2002).

Other demographic factors, such as body mass, weight, American Society of Anesthesiologists status, and education level were evaluated in only a few studies and were found to be related to postoperative pain and/or analgesic consumption only in isolated studies (Ip et al., 2009).

## 5. Conclusion

Due to the importance of postoperative analgesia and also because morphine is the standard index for the effectiveness of drugs in pain control, this research tries to compare the analgesic effect and the side effects of paracetamol and morphine using PCA method. Paracetamol could be as successful as morphine in pain control and analgesia in the first postoperative hours and its effect increases with the passage of time. Although morphine seems to be more effective at 2 and 4 hours, but after 4 hours they have similar effects. With due attention to this and similarity of patient satisfaction in both groups Paracetamol could be considered as an alternative drug.
